# Fortified Iodine Milk Improves Iodine Status and Cognitive Abilities in Schoolchildren Aged 7–9 Years Living in a Rural Mountainous Area of Morocco

**DOI:** 10.1155/2016/8468594

**Published:** 2016-03-16

**Authors:** Fatima Ezzahra Zahrou, Mehdi Azlaf, Imane El Menchawy, Mohamed El Mzibri, Khalid El Kari, Asmaa El Hamdouchi, Fatima-Zahra Mouzouni, Amina Barkat, Hassan Aguenaou

**Affiliations:** ^1^Joint Unit of Nutrition and Food Research (URAC39), CNESTEN-Ibn Tofaïl University, Regional Designated Center for Nutrition (AFRA/IAEA), 14000 Kenitra, Morocco; ^2^Ministry of Health, Rabat, Morocco; ^3^Équipe de Recherche en Santé et Nutrition du Couple Mère Enfant, Faculté de Médecine et de Pharmacie de Rabat, Université Mohammed V de Rabat, Rabat, Morocco

## Abstract

Iodine is required for the production of the thyroid hormones essential for the growth and development of the brain. All forms of iodine deficiency (ID) affect the mental development of the child. Our study aims to assess the impact of ID on the intellectual development of Moroccan schoolchildren and to evaluate the effect of consumption of fortified milk on reducing ID. In a double-blind controlled trial conducted on schoolchildren, children were divided into two groups to receive fortified milk (30% of cover of RDI iodine) or nonfortified milk for 9 months. Urinary iodine was analyzed using the Sandell-Kolthoff reaction, a dynamic cognitive test using Raven's Standard Progressive Matrices to assess learning potential was performed at baseline and end line, and anthropometric assessment was done only at baseline. The study included schoolchildren who were severely iodine deficient. The prevalence of malnutrition was high in both groups; in this study, we found improvements in iodine status and in cognitive abilities among Moroccan schoolchildren. Our study showed that the consumption of fortified milk led to a clear improvement in iodine status and also appeared to have a favorable effect on the cognitive ability of Moroccan schoolchildren in a rural mountainous region.

## 1. Introduction

2 billions of world's population have insufficient iodine intake, and the majority of the world's children have iodine deficiency (ID) with higher rates in developing countries [1, 2]. And all degrees of ID (mild: iodine intake of 50–99 *μ*g/day, moderate: 20–49 *μ*g/day, and severe: <20 *μ*g/day) have many adverse effects on growth and mental development [3]. These effects are collectively termed iodine deficiency disorders (IDD). The damage increases with the degree of the deficiency, and the most serious adverse effect of ID is endemic cretinism [4]. These effects are due to inadequate production of thyroid hormone due to an insufficient iodine intake [1]. In Morocco, the severity of the IDD is considered as moderate; indeed a regional prevalence survey done in mountainous areas of Azilal in 1992 revealed that 65% of children examined were goitrous [5, 6]. Iodine deficiency results in a global loss of 10–15 Intellectual Quotient (IQ) points at a population level and constitutes the world's greatest single cause of preventable brain damage and mental retardation [3]. The relationship between iodine deficiency and early cognitive development has captured recent attention because iodine is related to specific physiological processes [7]. Studies on the effect of iodine deficiency on children's cognition and behavior are selectively reviewed, looking for evidence of a causal relationship [4, 8]. Most correlational studies have found associations between ID and poor cognitive and motor development and behavioral problems. Longitudinal studies consistently indicate that children having iodine deficiency in infancy continue to have poorer cognition, school achievement, and more behavior problems in middle childhood [4, 8]. However, the possible confounding effects of poor socioeconomic backgrounds prevent causal inferences from being made. In nearly all countries, the best strategy to control iodine deficiency is iodization of salt, which is one of the most cost-effective ways to contribute to economic and social development [1]. Nowadays, and after 20 years of salt fortification with iodine in Morocco, the results are far from being satisfactory [9]. International efforts to control iodine deficiency disorders are slowing down and reaching the third of the worldwide population that remains deficient is still a major challenge. Fortification of basic food such as dairy products was developed as an alternative approaches for combating IDD [10, 11]. Thus, in collaboration with the Foundation for Child Nutrition, a leading manufacturer in the distribution of dairy products in Morocco, we have undertaken the current study to evaluate iodine status and its effect on cognitive ability (learning potential) after consumption of iodine fortified milk among schoolchildren aged 7 to 9 years, living in rural mountain region of Morocco.

## 2. Materials and Methods

### 2.1. Study Design

This study is a longitudinal interventional, double-blind (participants and assessors), and controlled one conducted among Moroccan schoolchildren, aged 7–9 years between February and October 2012, in a rural mountainous region. A total of 200 schoolchildren were recruited from three primary satellite schools and were divided into two groups to receive daily 200 mL either the fortified or the nonfortified milk. A distance of 52 km separated the two sites to avoid errors of distribution and/or exchange of milk batches between schoolchildren. To be included in the study, children had to be aged between 7 and 9 years and should not take supplements during the study period. Children with severe malnutrition needing nutritional rehabilitation or having chronic or severe illness requiring hospitalization or treatment were excluded from the study (and transferred to a local health center for follow-up). The study was conducted with respect to ethical and legal aspects, and written informed consent was obtained from each parent of recruited children.

### 2.2. Sample Size

The calculation of sample size was based on the standard deviation (2.5 *μ*g/L) of the rate of urinary excretion of iodine [12]. To observe a difference of 2 *μ*g/L with 5% level of significance and 90% power between the intervention group and placebo and after accounting for 15% dropouts, sample size of 40 children per group was required [13].

### 2.3. Milk Composition

The milk used was developed and produced specifically for this study by the Foundation for Child Nutrition to meet the purpose of the survey. It was whole, flavored with vanilla and sterilized by Ultrahigh Temperature (UHT). Both fortified milk and nonfortified milk were identical in taste and smell; the containers had the same appearance and packaging and they were distributed in schools during the 9 months of this study (including weekends and vacation days) [9]. The quality and quantity of nutrients of each batch of milk were doubly checked by AQUANAL (Laboratoire Aquitaine Analyses) in France and LOARC (Laboratoire Officiel d'Analyses et de Recherches Chimiques de Casablanca) in Morocco before their use in the study.

### 2.4. Data Collection

At recruitment, data regarding socioeconomic status (SES) were administered to each child and anthropometric parameters were measured. Cognitive ability was evaluated dynamically and the random urine samples were collected in the morning to assess urinary iodine using the Sandell-Kolthoff reaction at baseline and after 9 months of intervention.

### 2.5. Socioeconomic Status Assessment

Data regarding SES were collected at the beginning of the study from parents in all subjects groups, using questionnaires including level of parental education, household size, and monthly alimentary expenses.

### 2.6. Anthropometric Measurements

Anthropometric measurements were taken following standard procedures [9, 14] at baseline. Underweight and stunting were defined as weight-for-age *Z*-scores (WAZ) and height-for-age *Z*-scores (HAZ) < −2, respectively, according to the World Health Organization (WHO) [15].

### 2.7. Psychometric Test

In our study, the cognitive ability was evaluated dynamically by using a dynamic procedure instrument using the nonverbal part of Standard Progressive Matrices of Raven (SPMR) as a starting point. The SPMR, used in our study, are suitable for children from the age of six [16]. Dynamic testing is intended to assess children's learning potential or ability to benefit from instruction (Raven test-intervention-retest paradigm). The dynamic tests look at people's ability to learn while they are being tested [17]. This Kind of testing procedure involves an initial test without instruction and then a pretest which provides data on the children's current level of functioning; an instruction component is then given with the aim of familiarizing children with test demands, equalizing their experiences, and teaching the necessary problem solving skills; and finally the student's new level of functioning is tested during a posttest session. The intervention phase should give children, who did not have an adequate opportunity to develop their academic potential, a greater chance to achieve a fair test result.

The opening test consisting of set A of SPMR (items A1 to A12) was used to introduce children to the test material; the first two items of test 1 were used as items of examples. Test 2 (pretest and posttest) was built based on the items in sets B and C of SPMR (items B1 to B5, B7, B9, and B11; items C1 to C4, C6, C8, C10, and C12); the first two items of test 2 were also used as items of examples. Instruction component (test 3) was performed using the items B6, B8, B10, C5, C7, C9, and C11; the evaluators gave standardized explanations on how to solve the problems to all children (using a paper-pen support, drawing in blackboard, and giving explanations to children one by one).

The test protocol was translated into Moroccan dialect. Two health workers were trained to conduct the test which was pretested on a homogenous sample of *n* = 48 children (not included in the study) to ensure appropriateness of the test materials, to check item difficulties, and to standardize the passing protocol, especially the instruction phase. Instructions were explained to the children in Moroccan dialect and local Berber dialect (tachelhit). The passing of the test was made collectively in small groups of half-classes in a class room which was quiet and free of distractions; the maximum duration of the dynamic assessment was 1 hour and 30 min.

### 2.8. Urine Sampling

Random urine samples were collected in the morning, between 10 a.m. and 11 a.m. to assess urinary iodine [18]. These samples were aseptically collected in 40 mL capped polypropylene tubes, aliquoted in 4 mL Cryovial tubes, and stored at −20°C until analysis. Urinary iodine was determined spectrophotometrically using the Sandell-Kolthoff reaction [19]. According to the level of iodine in urine, the iodine deficiency is classified into three classes; normal iodine status: >100 *μ*g/L; mild iodine deficiency: 50–99 *μ*g/L; moderate iodine deficiency: 20–49 *μ*g/L; and severe iodine deficiency [20].

### 2.9. Statistical Analysis

Data analysis was done by the software IBM SPSS Statistics version 20 (Statistical Package for the Social Sciences). Anthropometric *Z*-scores were calculated using WHO standards. The distribution normality of the quantitative variables was tested by Kolmogorov-Smirnov test. The variables normally distributed were presented as mean ± standard deviation. The nominal variables were presented as proportion and 95%. Chi-square test was used to test independence between nominal variables. In the case of cells with a theoretical frequency *n* < 5, we take the *p* value of Fisher. Two-sided *p* values < 0.05 were considered significant.

For cognitive tests, Quade's rank-transformed analysis of covariance was used [16], linear regression of the ranks of the 9th month posttest score was run on the ranks of the covariates (baseline posttest score, baseline urinary iodine), then the unstandardized residuals were saved, and finally a one-way analysis of variance was run using as a dependent variable, the unstandardized residuals of the ranks of the 9th month posttest score and the grouping factor (fortified milk/nonfortified milk) as the factor. The linearity was assessed and homogeneity of variance was met with Levene's test.

## 3. Results

The rural and mountainous region where the study was conducted is characterized by a community with low to medium income, more than half of parents being analphabet [9], and a relatively high prevalence of iodine deficiency [21]. Furthermore, over a third of children in the region suffer from stunting knowing that the national prevalence is 14.9% [22]. The mean age of the studied population is 8.0 ± 6.7 years with sex ratio 1.10.

**Figure 1:**
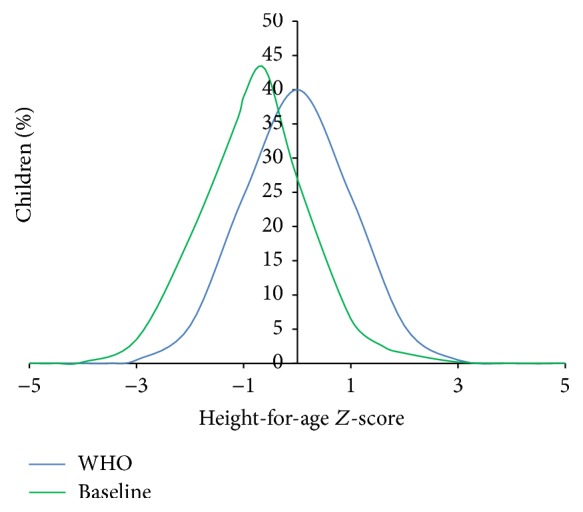
Distribution of the index height-for-age in schoolchildren studied at baseline compared to the reference population (WHO).

Figure 1 shows the distribution of the anthropometric index height-for-age in schoolchildren 7 to 9 years compared with the reference population. The analysis of the figure shows that 8.5% of children are stunted (HAZ score < −2 SD). The distribution of the ratio height-for-age among children examined is moved to the left and is below the median compared to the distribution of the reference population (Figure 1).

**Figure 2:**
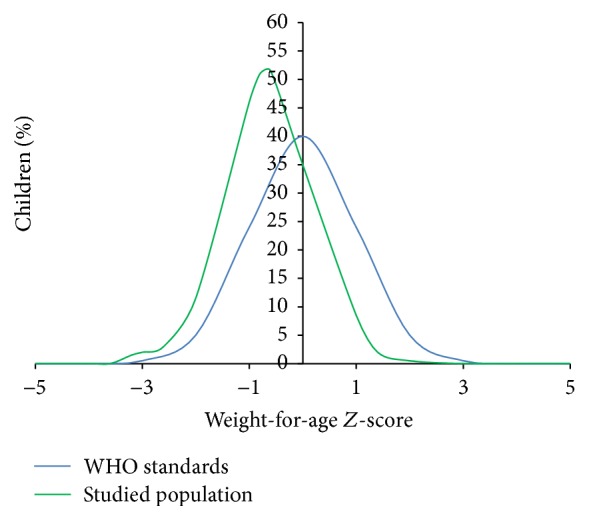
Distribution of the index weight-for-age in schoolchildren studied at the beginning of the study compared to the reference population (WHO).

Figure 2 shows the distribution of the anthropometric index weight-for-age in children examined aged 7 to 9 years compared with the reference population. The analysis of the figure shows that 3.4% of children are underweight (WAZ score < −2 SD). We also noted that our population is shifted to the left, thus being below the median of the reference population (Figure 2).

**Figure 3:**
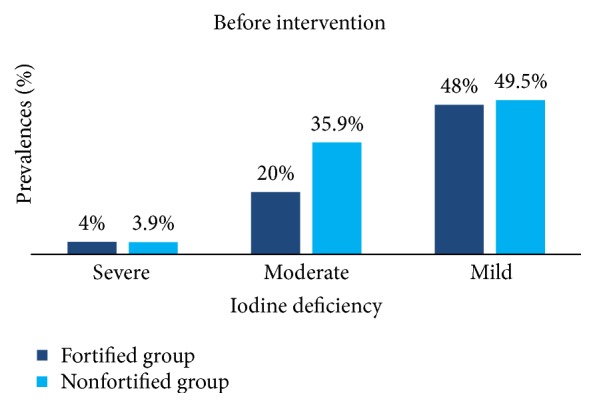
Prevalence of iodine deficiency among both groups before intervention.

Figure 3 illustrates the prevalence of severe, moderate, and mild iodine deficiency among fortified and nonfortified groups at the beginning of the survey. We observed that before intervention the studied groups have approximately the same level of different classes of iodine deficiency and no statistical difference was registered between groups (*χ* ^2^-test, *p* > 0.05).

**Figure 4:**
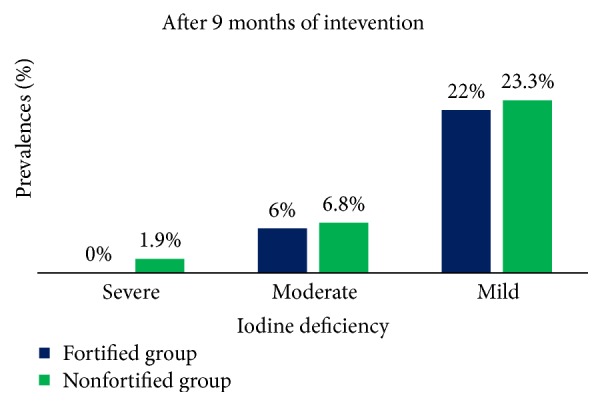
Prevalence of classes of iodine deficiency among fortified and nonfortified milk groups after 9 months of consumption of milk.

Figure 4 indicates that after 9 months of consumption of fortified or nonfortified milk among Moroccan schoolchildren the status of severe, moderate, and mild iodine deficiency was improved.

The results of dynamic cognitive test scores after receiving either iodine fortified milk or noniodine fortified milk are shown in Table 1. The results from Quade's test after controlling for confounding variables showed a significant *p* value between the fortified group and the nonfortified group at the end of the study, with the fortified group being better (*p* = 0.020).

**Figure 5:**
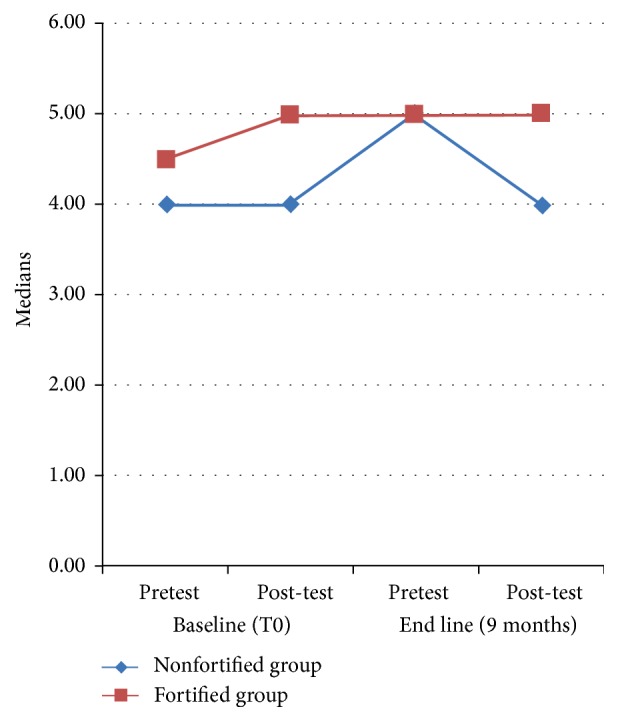
Comparison of cognitive test scores between the two studied groups.

The comparison of cognitive test scores between the two study groups showed an improvement of learning abilities in favor of the fortified group over 9 months of intervention period (Figure 5).

## 4. Discussion

The study was designed to investigate whether regular consumption of multiple micronutrients fortified milk over a period of 9 months could improve the iodine status and the effects of iodine deficiency on school children's cognitive abilities. To our knowledge, this is one of the first studies that used milk as a vehicle for delivery of micronutrients and evaluated the impact among school children in a double-blind Randomized Controlled Trial (RCT) in Morocco. After 9 months of intervention, our results showed that the consumption of milk fortified with potassium iodide and other micronutrients is efficacious in reducing the prevalence of iodine deficiency and improving iodine status indicators in a sample of 7–9-year-old children. The increase in the control group at the end of the study can be explained by the fact that the unfortified milk also contained some iodine. A systematic review [23] evaluated the effect of the multimicronutrient (MMN) fortification of foods compared to unfortified foods on the micronutrient status of school children and measured a statistically significant improvement in iodine status in the intervention group compared to the control group, taking baseline values into account. In South Africa, fortified biscuits resulted in a significant improvement in the iodine status of primary school children from a poor rural community after 12 months of intervention [24]. A study conducted in Filipino schoolchildren showed that consumption of a multiple-micronutrient-fortified beverage for 16 weeks had significant effects on iodine status.

On the other hand, the link between iodine deficiency and cognitive development is direct but can be prevented through public health methods, making iodine deficiency the most preventable cause of mental retardation in the world [25]. In Morocco, there is no interventional study that has evaluated the effect of a school based micronutrient fortification on biochemical status as well as functional health outcomes including cognitive performances/abilities. Very few studies in Morocco have assessed the cognitive performances of school children in static way [26, 27]. The results of our study showed better effect for the iodine fortified milk group as compared with the nonfortified iodine milk group for dynamic testing after 9 months of intervention. The beneficial effect may have been due to improvements in myelination of central nervous system, particularly in the frontal cortex which is responsible for higher-order cognition and fluid intelligence, mediated by an increased supply of thyroid hormone [28] or by effects on neurotransmitters and/or glucose metabolism through better thyroid function [29].

In a meta-analysis of 18 observational studies, which compared children based on whether they lived in an iodine-deficient area or not, children who lived in iodine-deficient areas had deficits in cognitive functioning [30]. In a well-controlled observational study in Bangladesh, investigators found that children with mild hypothyroidism had deficits in spelling and reading compared to healthy controls [31]. Although evidence from these studies is compelling, families who live in iodine-deficient areas are often more impoverished than families in areas where iodine status is adequate. Similarly, in two placebo controlled, double-blind interventional trials in Albania and New Zealand [32, 33], increasing iodine intakes over several months improved cognition in school-aged children who presumably grew up under conditions of iodine deficiency. In Albania, moderately iodine-deficient 10–12-year-old children were randomized to receive either 400 mg of iodine as oral iodized oil or 400 mg of placebo for 6 months. Treatment with iodine improved iodine status and significantly improved information processing, fine motor skills, and visual problem solving [32]. In New Zealand, 10–13 y children were given a daily tablet containing 150 *μ*g iodine or placebo for 28 wk. Iodine improved scores on picture concepts and matrix reasoning [33]. In Filipino schoolchildren, salt iodization, accompanied by adequate intakes of energy, protein, and foods rich in thiamin and riboflavin, contributed to improved mental performance assessed by psychomotor and cognitive function tests (Bender-Gestalt and Raven's Colored Progressive Matrices) [34]. Furthermore, many observational studies have compared children in iodine-sufficient and iodine-deficient areas and nearly all have found poorer psychomotor or cognitive development in children living in iodine-deficient areas [35]. Iodine-deficient areas are generally more remote, poorer, and lacking in facilities compared with iodine-sufficient areas and these differences themselves could account for the children's poor development. Some authors have raised the problems of assessment of subclinical effects of ID and recommended the need for dynamic psychometric methods that reflect the contribution of sociocultural variables and skills acquired by learning to measure learning ability for assessing ID effect on the learning potential of children in endemic areas [36, 37]. However, information on iodine deficiency effect on children's learning potential in endemic areas remains rare [36].

## 5. Conclusion

We showed that milk can be used successfully as a vehicle for nutrient fortification in school feeding programs. Consumption of fortified milk resulted in a significant improvement in iodine status and also appeared to have a favorable effect on the cognitive ability of Moroccan schoolchildren in a rural mountainous region. The high prevalence of ID in the school aged population from poor rural background should be of concern and underlines the compelling need for implementing corrective and preventive measures to fight against this deficiency in school aged children from disadvantaged areas in Morocco.

Our study has already determined the feasibility and the efficacy of a geographical and targeted fortified school milk program in reducing the prevalence of ID among schoolchildren. Dynamic testing seems to be more appropriate for assessing children with poor educational backgrounds and could help to better detect the micronutrient fortification effect on cognitive ability.

## Additional Points

The major limitations of the study are the small size of the study population due to recruitment of children from only 3 schools.

## Figures and Tables

**Table 1:** Analysis of covariance table for posttest scores at the end line.

	Sum of squares	df	Mean square	*F*	*p* value^*∗*^
Between groups	47164.297	1	47164.297	5.564	0.020
Within groups	1356334.459	160	8477.090		
Total	1403498.756	161			

^*∗*^ *p* value was calculated using Quade's test.

## Abbreviations

ID: Iodine deficiency
IDD: Iodine deficiency disorders
IQ: Intellectual Quotient
UHT: Ultrahigh Temperature
AQUANAL: Laboratoire Aquitaine Analyses
LOARC: Laboratoire Officiel d'Analyses et de Recherches Chimiques de Casablanca
SES: Socioeconomic status
SPMR: Standard Progressive Matrices of Raven
SPSS: Statistical Package for the Social Sciences
FG: Fortified group
NFG: Nonfortified group
RCT: Randomized Controlled Trial
MMN: Multimicronutrients.
